# Dynamic learning and memory, synaptic plasticity and neurogenesis: an update

**DOI:** 10.3389/fnbeh.2014.00106

**Published:** 2014-04-01

**Authors:** Ales Stuchlik

**Affiliations:** Institute of Physiology, Academy of Sciences of the Czech RepublicPrague, Czech Republic

**Keywords:** learning, memory, behavior, synaptic plasticity, adult neurogenesis, hippocampus

## Abstract

Mammalian memory is the result of the interaction of millions of neurons in the brain and their coordinated activity. Candidate mechanisms for memory are synaptic plasticity changes, such as long-term potentiation (LTP). LTP is essentially an electrophysiological phenomenon manifested in hours-lasting increase on postsynaptic potentials after synapse tetanization. It is thought to ensure long-term changes in synaptic efficacy in distributed networks, leading to persistent changes in the behavioral patterns, actions and choices, which are often interpreted as the retention of information, i.e., memory. Interestingly, new neurons are born in the mammalian brain and adult hippocampal neurogenesis is proposed to provide a substrate for dynamic and flexible aspects of behavior such as pattern separation, prevention of interference, flexibility of behavior and memory resolution. This work provides a brief review on the memory and involvement of LTP and adult neurogenesis in memory phenomena.

## Introduction

The brain, with billions of cells’ connections and plethora of cell types in mutual interactions, is one of the most complicated organs in the human body and attracts attention of both scientific researchers and the public. This is underlined by the fact that we do not know the pathophysiology of many devastating brain disorders, which often affect memory and cognition (such as schizophrenia, Alzheimer’s disease etc.). Notably, the brain definitely does not work as an “elevated supervisor”; indeed, researchers working in neuroscience have now started to appreciate the “systems-level” understanding of the human body function in health and disease (Qureshi and Mehler, 2013).

Studying detailed physiology of any living system without evaluating its output (behavior), cannot give us enough information for understanding the function of the system. *Vice versa*, studying behavior without looking at the proximate mechanisms cannot provide sufficient insight, although striking exceptions from physiology exist such as ant navigation (e.g., Wohlgemuth et al., 2001), when scientists proceeded from studying behavioral outputs downstream to physiological mechanisms. Many scientists concerned with the integration of molecular and cellular views with systems-level and behavioral studies focus on the learning and memory. This particular function of nervous system in animals is well accessible by methods with different resolution (from spikes to molecules and cells to the whole organism).

In this review, I will try to provide a short update on integration of memory formation with a concept of synaptic plasticity (Hebb, 1949) (mainly long-term potentiation (LTP; Bliss and Lomo, 1973)) and adult neurogenesis in the dentate gyrus (DG) of the hippocampus (Altman, 1963; Altman and Das, 1965). My selection must obviously be subjective; there are excellent reviews on other issues related to this topic, such as the role of transcriptional factors and various kinases (Kandel, 2012; Xia and Storm, 2012). The rationale for selecting these subtopics settles on prevailing view on synaptic plasticity processes as a “hotspot” of current research into basic mechanisms of learning and memory (Glanzman, 2013). Focus on neurogenesis is based on the fact that despite many memory studies involve static settings, our world is endlessly dynamic and learning should be considered a highly dynamic process; neurogenesis may provide these flexible aspects of memory. These fields open ways for searching mechanisms of deficits in many neuropsychiatric disorders with enormous human and socioeconomic impact and suggest ways of novel causal therapies for depression, PTSD, Alzheimer’s disease or schizophrenia (Voineskos et al., 2013). Scientists relate both LTP and neurogenesis to memory (Brown et al., 1990; Snyder et al., 2001), LTP as a candidate mechanism for long-term retention of information and hippocampal neurogenesis as a candidate mechanism for specific dynamic and flexible aspects of learning.

## Learning and memory

Memory refers to a capability of virtually any animal to encode, store and retrieve information, to guide behavioral output. Learning is viewed as acquisition or encoding the information to memory. It is an excellent example of model system allowing for multi-level analysis (again, note close relation of memory deficits to these pathological conditions). Absence/presence of a memory trace (engram) is often presented or even defined as a change of a particular behavior (such as a rat with lesions to the hippocampus may get lost in a spatial maze), or then, as a change of ability to learn/remember. The term engram was first coined by Richard Semon, a German biologist (Semon, 1921). It usually refers to mechanisms (or tags) by which the memories are stored. The prevailing view today is that memory should leave physical (e.g., molecular) and often rather distributed changes in neuronal tissue (Moser and Moser, 1998; Frey and Frey, 2008).

Another well-accepted opinion is that there is nothing like “universal memory”; instead, multiple memory systems exist (Doeller et al., 2008; Lee et al., 2008; Schwabe, 2013) having their specific (partially competing but also shared) brain resources to fulfill their tasks (Squire, 2004). Temptating is a concept that memory may be stored (at least in mammals) in distributed changes in synaptic weights, which may modulate synchronization and grouping of firing of neuronal assemblies (Hebb, 1949; Harris et al., 2003). Further introspection of the engram and its nature also provokes many questions about stability, localization, time course of possible changes, and consolidation and transformation of a memory trace (reviewed in Dudai, 2004).

Importantly, complex and vulnerable mammalian memory types, i.e., declarative and spatial memory, depend on the medial temporal lobes (MTL) of the brain (reviewed in Eichenbaum, 2001). These are the hippocampus, subiculum and neighboring cortical areas such as entorhinal, perirhinal and postrhinal cortices (Amaral and Witter, 1989). Many scientist today are convinced that the hottest candidates for neural correlate of a long-term memory trace are long-term changes in synaptic strengths (Hebb, 1949), i.e., LTP and long-term depression (LTD). For the sake of simplicity, this minireview selects LTP out of the synaptic plasticity mechanisms, although there is a great evidence for LTD role in learning and memory as well (reviewed in Kemp and Manahan-Vaughan, 2007).

### LTP, protein kinase Mzeta and memory

Already in 1973, Terje Lomo a Timothy Bliss published a seminal study (Bliss and Lomo, 1973) showing that tetanization of specific pathway in the hippocampal formation of anesthetized rabbit resulted in significant increase in the excitatory postsynaptic potentials in postsynaptic cells, which further supported Hebb’s theories (Hebb, 1949). Later on, this phenomenon was demonstrated in anesthetized and freely moving rats and mice. Today, LTP attracts many scientists, because it represents an intriguing but artificial model of long-term changes of the CNS function (more than 12,000 hits in PubMed on the term search). However, causal link between LTP and memory has been suspected but not settled for a long time, although supportive studies were provided earlier, in which interference with LTP affected learning and memory.

Multiple studies have demonstrated that interference with NMDA and AMPA receptor function (e.g., Steele and Morris, 1999; Bast et al., 2005) blocks certain phases of LTP and memory. Importance of NMDA receptors was documented especially in one-trial learning, such as in the delayed-matching to place version of the Morris water maze (MWM), a classical spatial task (Steele and Morris, 1999). Other studies used a different approach, i.e., tetanization of the majority of hippocampal synapses and subsequent testing in hippocampus-dependent task. Initially, it was shown that such LTP saturation disrupts subsequent spatial memory in the MWM probe trials (Moser and Moser, 1999), but this effect has been found to be eliminated by non-spatial pretraining (Otnæss et al., 1999), suggesting that LTP induction may provide a mechanism for capturing the proper strategy in the task. Despite controversies, all these studies corroborated the hypothesis that LTP has some relation to learning and memory.

In 2006, two studies convincingly reported the link between LTP and learning and memory. Whitlock et al. (2006) examined the hypothesis that not only tetanization but also memory encoding *per se* may induce long-term plastic changes. The study showed that training rats to solve inhibitory avoidance, in which animal learns to avoid a preferred dark compartment punished by mild footshocks led to induction of the LTP in a subset of hippocampal synapses. Some synapses were unaffected, again supporting the concept of distributed memory trace. It is interesting to note that inhibitory avoidance learning, despite a simple paradigm, contains both operant and contextual fear conditioning component and involves highly coordinated recruitment of molecular and cellular machinery in the hippocampal formation (Izquierdo et al., 2002). The paper by Whitlock and colleagues contributed much to the notion that memory encoding may produce LTP in some synapses and that memory acquisition could be analogized to electrical tetanization of the synapse. Other studies have strongly corroborated this view (Cohen et al., 2011; Rodríguez-Durán et al., 2011; Kenney and Manahan-Vaughan, 2013).

Another paper in the same issue of Science (Pastalkova et al., 2006) focused on the maintenance phase of LTP as a candidate mechanism for memory storage, based on previous robust evidence from the laboratory of Todd Sacktor that an atypical form of protein kinase C (PKMzeta) is necessary and sufficient for maintenance phase of the LTP (Ling et al., 2002). The study employed so-called zeta-inhibiting peptide (ZIP), which was injected into hippocampi of rats that previously acquired the spatial active place avoidance task. Subsequently, memory retention was tested and it was found selectively impaired by ZIP injection. Interestingly, such microinjection failed to abolish novel learning in the same task, suggesting that PKMzeta erased previous memory trace but did not block encoding of novel information.

Subsequently, Shema et al. (2007) have shown that injection of ZIP into the insular cortex of the rat erased conditioned taste aversion, an evolutionary advantageous type of conditioning. This memory is traditionally measured by avoidance of ingestion of a food, the flavor of which had been associated previously with sickness (Buresová et al., 1979). Another study has corroborated these findings by extension to erasure of other types of memory, such as classical or instrumental conditioning (Serrano et al., 2008). Injection of ZIP also reduced the precision of the MWM representation in the probe trial, despite the rough localization of the goal was still present. Additional evidence on PKMzeta and memory storage came from a recent study (Shema et al., 2011), which showed that overexpression of PKMzeta in the insular region enhanced the conditioned taste aversion memory. Moreover, Pauli et al. (2012) has revealed that blockade of PKMzeta also has an effect in the striatum, affecting instrumental response selection and habits.

However, Volk et al. (2013) have recently generated constitutive and conditional PKMzeta-knockout mice and detected no impairment of either LTP maintenance or hippocampus-dependent memory (Volk et al., 2013). Since the previous reports sometimes used pharmacological blockade of enzyme by ZIP, Volk et al. also applied ZIP to their transgenic mice lacking PKMzeta and detected LTP suppression. This suggests ZIP targets other enzymes required for LTP. Analogous results have been found by Lee et al. (2013) in PKMzeta-null mice. The role of PKMzeta has therefore been questioned (Kwapis and Helmstetter, 2013).

Interestingly, another form of PKC named iota (Selbie et al., 1993) was suggested to compensate for deficient PKMzeta in these experiments, suggesting that more diverse cascade can provide basis for memory maintenance rather than a single “memory molecule” (Glanzman, 2013). In any case, scientists appear to be on the track of interesting discovery of how our vivid everyday memories relate to molecular and cellular brain processes.

### Adult neurogenesis in the dentate gyrus and memory

Importantly, the hippocampus, specifically the subgranular zone of the DG, is one of two sites of neurogenesis in the adult brain (Altman, 1963; Altman and Das, 1965). Some of the dividing neural progenitor cells survive, differentiate into neurons and incorporate into the hippocampal network. The physiological, especially the behavioral role of adult neurogenesis is a subject of controversies until today. Newly born neurons in the DG are proposed to facilitate learning in the hippocampus by separating overlapping patterns in hippocampal inputs, thus ensuring formation of distinct representations.

Enhancing adult neurogenesis in the DG was found to suffice for improvement of pattern separation (Sahay et al., 2011) and another recent study (Nakashiba et al., 2012) suggested that newly-born hyper-excitable neurons may participate preferentially in pattern separation, while older adult granule neurons provide mainly pattern completion (a complementary process which allows adding missing features into incomplete hippocampal representations). This study did not focus directly on the autoassociative network in CA3, but in light of paper by Rolls (2013), I propose that strong, “consolidated” synapses between older granule neurons via mossy fibers to CA3 may provide a significant contribution to pattern completion. It should be noted that a precise balance between pattern completion and pattern separation is probably one if the functions of CA3 upstream region innervated by mossy fibers from dentate granule cells, which mediates a proper hippocampus function and such interplay may be disrupted in memory disorders (Hanson and Madison, 2010).

Another branch of research on the functional role of adult neurogenesis has proposed that adult neurogenesis in the hippocampus may also ensure prevention of interference of new memories with old ones (Wiskott et al., 2006). A study using olfactory memory task demanding interference resolution has also supported such role for new granule neurons in the DG (Luu et al., 2012). A recent study by Gordon Winocur et al. have strongly supported this prediction (Winocur et al., 2012) using a visual discrimination task under conditions of low or high interference. A very recent study by Déry et al. (2013) confirmed such role of neurogenesis even in humans and shown a positive impact of voluntary exercise and adverse effects of depression. Recently, the role of DG adult neurogenesis has been extended to increase “memory resolution” so that cooperation between newly born, hyperexcitable granule cells and older neurons that code sparsely for salient features increases the amount of detail encoded in hippocampal memories (Aimone et al., 2011).

Studies also showed that hippocampal neurogenesis promotes behavioral flexibility in mice. A study by Garthe et al. (2009) demonstrated the subtle but significant effects of neurogenesis ablation. Using chronic treatment with a cytostatic temozolomide and efficient recovery protocol, the memory differences between mice with and without adult neuronal proliferation were revealed in the MWM not by traditional measures such as distance to reach the platform, but by evaluating spatial/non-spatial strategies possessed in the maze. Robust evidence on behavioral flexibility account of neurogenesis has been provided by Burghardt et al. (2012), who showed that neurogenesis ablation in mice led to impairments of reversal learning in active place avoidance task (reviewed by Stuchlik et al., 2013). It also disrupted flexible incorporation of second reference frame (second to-be-avoided place, i.e., two-frame place avoidance; reviewed in Stuchlik et al., 2013). The deficit could not be explained by alteration of memory extinction, or with inability to acquire new memory in a novel environment; these functions were spared in both groups. Interestingly, the flexibility impairment was accompanied by immediate-early gene *Arc* up-regulation, suggesting effects on excitability and plasticity of the hippocampal network (Pevzner et al., 2012).

Recently, time-limited role of new, hyper-excitable granule neurons in the DG in hippocampus-dependent memory has been shown by optogenetic approach (Gu et al., 2012). This study showed that newly born cells form functional synapses on pyramidal neurons of CA3 region from 2 weeks after their birth reaching a stable state at 4 weeks. Newborn neurons at this age were more plastic than neurons at other stages. The study also showed that switching off this cell type of 4-week-old cells after encoding disrupted retrieval of hippocampal memory. This suggests that these 4 weeks represent a functional time window for adult-born neurons in hippocampus-dependent memory retrieval (Gu et al., 2012). Using multiple high-resolution methods, a study of Ikrar et al. (2013) clearly documented that blocking adult neurogenesis increased excitability in the DG networks while its enhancement has reduced it, and also pointed to an important role of inhibitory GABAergic interneurons (Ikrar et al., 2013).

Based on this, we propose that neurogenesis in the hippocampus might underlie so-called behavioral separation, which we define as selective recruitment of distinct and dissociable hippocampus-functions such as spatial representation and cognitive coordination (Kubík and Fenton, 2005; Wesierska et al., 2005). Such role might be shown by selective neurogenesis ablation and testing in massed/alternate protocols using the specific behavioral tasks (MWM, Morris, 1981) and active place avoidance on Carousel (Stuchlik et al., 2013). Additionally, newly born neurons may contribute to coping with dynamic, changing aspects of memory (such as with moving goals) in accordance with the evolutionary hypothesis of neurogenesis role (Kempermann, 2012).

## Short notes on some other factors affecting LTP, neurogenesis and memory

There are additional factors affecting memory, LTP, and neurogenesis. For instance, a recent study on human glial cells implanted into mouse hippocampus documented enhanced LTP, suggesting strong functional role of this cell type in the brain (Zhang and Barres, 2013). Neurogenesis is also significantly affected by ageing, stress and disease but contrarily enhanced e.g., by enriched environment (Kempermann et al., 1997) and physical exercise (van Praag et al., 1999). Generally, all these phenomena in parallel affect memory as well (Nilsson et al., 1999; Lithfous et al., 2013). An intriguing study by Van der Borght et al. (2007) showed beneficial effects of physical exercise and dietary restriction on spatial T-maze performance and surprisingly, T-maze learning and reversal training actually led to reduced neurogenesis, suggesting optimal balance of these processes for proper memory maintenance.

Of high importance is the role of sleep in memory (McCoy et al., 2013). It has been shown that during both non-REM and REM sleep cellular processes may take part, subserving memory (Benington and Frank, 2003). A factual necessity of sleep for forming and consolidation of memories has emerged recently (Prince et al., 2014). Sleep is also related to neurogenesis (Mueller et al., 2013) as well as to LTP (Kim et al., 2005). From a pharmacological point of view, a recent study have shown that anti-diabetes drug metformin might offer a promising way of increasing adult neurogenesis and memory function (Wang et al., 2012) that is impaired in several neuropsychiatric disorders.

## Conclusions

Much evidence converges on the view that learning and memory, synaptic plasticity and neurogenesis are inter-related phenomena. Specifically, the latter two are considered to provide substrate for specific aspects of learning and memory function. LTP maintenance probably underlies memory retention and its inhibition erases memory. Encoding of a memory trace induces LTP in subset of hippocampal synapses. Neurogenesis underlies specific dynamic and flexible features of learning and memory phenomena in the precisely regulated and time-restricted manner. Nowadays and in the future, collaborative and multi-disciplinary efforts involving optogenetics, transgenes, *in vivo* patch-clamp etc. will bring significant insight into mechanisms of memory.
